# Aortic Valve Phenotype Shapes Helical Flow in the Ascending Aorta: Insights from 4D Flow MRI

**DOI:** 10.1007/s10439-026-04088-8

**Published:** 2026-04-07

**Authors:** Karol Calò, Andrea Guala, Valentina Mazzi, Lydia Dux-Santoy, José F. Rodriguéz Palomares, Stefania Scarsoglio, Luca Ridolfi, David A. Steinman, Diego Gallo, Umberto Morbiducci

**Affiliations:** 1 PolitoBIOMed Lab, Department of Mechanical and Aerospace Engineering, Politecnico di Torino, Turin, Italy; 2 Vall d’Hebron Institut de Recerca, Barcelona, Spain; 3 Biomedical Research Networking Center on Cardiovascular Diseases, Instituto de Salud Carlos III, Madrid, Spain; 4 Department of Cardiology, Hospital Universitari Vall d’Hebron, Barcelona, Spain; 5 PolitoBIOMed Lab, Department of Environment, Land and Infrastructure Engineering, Politecnico di Torino, Turin, Italy; 6 Department of Mechanical and Industrial Engineering, University of Toronto, Toronto, Canada

**Keywords:** 4D flow MRI, Ascending aorta, Bicuspid aortic valve, Helical flow, Hemodynamics, Helicity

## Abstract

**Purpose:**

Helical flow in the ascending aorta (AAo) is recognized as beneficial to cardiovascular physiology. Previous in vivo studies of bicuspid aortic valve (BAV) disease have mainly relied on qualitative assessments or surrogate measures of helical flow, hampering its use as potential hemodynamic biomarker. Here, we leveraged a rigorous fluid mechanical framework for helical flow structures to (i) determine how aortic valve (AV) phenotype—tricuspid (TAV) versus bicuspid—influences AAo helical flow and (ii) identify its principal anatomical and hemodynamic determinants.

**Methods:**

4D flow MRI data from sixty subjects (41 TAV, 19 BAV) were analyzed. Helicity-derived quantities were computed to quantify the intensity and topology of AAo helical flow. Conventional hemodynamic and anatomical parameters were also extracted and their association with helicity-based quantities was explored.

**Results:**

Compared with TAV subjects, BAV patients exhibited lower helicity intensity and reduced predisposition to form coherent helical flow patterns. In TAV, helical flow topology was primarily influenced by flow pulsatility and vessel anatomy, whereas in BAV, eccentric systolic jets promoted the arrangement of blood flow into helical structures without enhancing helicity intensity. Helical flow topology emerged as highly sensitive to AV phenotype, with its discriminative power augmented by conventional anatomical or hemodynamic parameters.

**Conclusion:**

AV phenotype critically shapes helical flow in AAo through phenotype-specific anatomical and hemodynamic determinants. The eccentric jet associated with BAV disrupts helicity intensity, potentially diminishing the protective role of helical flow. Integrating conventional hemodynamic and anatomical parameters with helical flow topology yields a robust, in vivo measurable morpho-hemodynamic signature of BAV, offering diagnostic and prognostic potential.

**Supplementary Information:**

The online version contains supplementary material available at https://doi.org/10.1007/s10439-026-04088-8.

## Introduction

Starting in the mid-1980s, in vitro experiments with curved and branching models demonstrated the presence of coherent helical flow patterns in large arteries [1–3]. These findings were later confirmed by both in silico and in vivo studies [4–10]. Today, helical flow is widely recognized as a physiologically significant feature of the cardiovascular system [11]. This phenomenon is particularly prominent along the human aorta [5, 12–16], where it plays a central role in maintaining vascular health. Compelling evidence indicates that distinct helical flow patterns exert an atheroprotective effect by attenuating flow disturbances—commonly quantified in terms of atherogenic wall shear stress (WSS) [8, 17]—and enhancing momentum and mass transport, thereby reducing the residence time of circulating pro-atherogenic particles [11, 18]. Furthermore, consistent with fluid mechanics theory [19], the organization of aortic flow into coherent helical structures promotes flow stability and delays or even prevents the transition to turbulence [13, 14, 20].

The recognition of the physiological significance of helical flow in the aorta has driven the adoption of advanced imaging modalities for its detailed characterization in vivo. Among these, 4D flow MRI has emerged as the non-invasive gold standard for assessing aortic hemodynamics [21]. This technique enables the simultaneous acquisition of anatomical information and time-resolved velocity vector fields, providing detailed insights into complex fluid dynamics. Despite these advances, many studies have relied on qualitative or semi-quantitative grading approaches to characterize aortic flow in terms of helicity content. These often involve visual inspection of instantaneous velocity streamlines—which can be misleading from a fluid mechanics perspective (see discussion in [22, 23])—or particle tracking of elemental fluid volumes (fluid pathlines) [22, 24–26]. While informative, such qualitative approaches are inherently observer-dependent and provide only a limited description of the aortic helicity. To address these limitations, objective and quantitative metrics have been proposed to capture both intensity and topology of helical flow, thereby enabling rigorous volumetric characterization within the vascular bed of interest [7, 13, 27]. Several of these quantitative approaches have already been successfully applied to 4D flow MRI data to investigate helical flow in both healthy and diseased aortas [14, 28–32].

Research on aortic helical flow has been particularly relevant in the context of bicuspid aortic valve (BAV) disease, a common congenital cardiac defect which affects 1–2% of the population and predisposes patients to serious complications such as ascending aorta (AAo) dilation, aortic valve (AV) stenosis, and aortic dissection. Notably, several studies have reported abnormal AAo flow patterns in BAV patients, even in the absence of concomitant aortic dilation or AV stenosis [28, 29, 33–38]. These flow disturbances have been attributed to the asymmetric geometry of the BAV orifice—unlike the more symmetric tricuspid AV (TAV)—which produces eccentric, high-velocity systolic flow jets that impinge on the aortic wall. Such flow jets may profoundly disrupt AAo hemodynamics [37, 39], leading to abnormal WSS distributions and unphysiological, eccentric helical flow structures. Both phenomena are regarded as key drivers of BAV-associated aortopathy [26, 40–43].

Given the well-established physiological significance of helical flow and the high prevalence of BAV-related complications, the present study aims to investigate the impact of AV phenotype on AAo helical flow in vivo using 4D flow MRI. To address the limitations of previous in vivo studies, which largely characterized aortic helical flow only partially—either via visual inspection, or on 2D analysis planes [29] or by quantifying surrogate measures of helicity [44–47]—we leveraged a rigorous theoretical framework defining helical flow in terms of kinetic helicity density and helicity as the degree of knottedness of tangled vortex lines [48, 49]. Technically, we analyzed the topology and intensity of helical flow in vivo by computing established helicity-derived quantities [7, 13, 27] on aortic 4D flow MRI datasets. We hypothesized that AV phenotype critically modulates both the intensity and topological organization of AAo helical flow. Specifically, we postulated that the BAV phenotype disrupts the formation of coherent, organized helical fluid structures and is associated with reduced helicity intensity, in contrast to the physiological helical flow patterns characteristic of healthy TAV individuals. We further hypothesized that such alterations may compromise the protective effects of helical flow, thereby contributing to the pathophysiology of BAV-associated aortopathy. The primary objectives of the analysis were, therefore, (i) to identify the major anatomical and hemodynamic determinants of AAo helicity in subjects with TAV and BAV phenotypes and (ii) to assess whether distinct helical flow characteristics could provide additional value in defining a more robust combined morpho-hemodynamic signature of BAV disease.

## Materials and Methods

### Study Population

A total of 107 adult participants from a previous cross-sectional cohort study [35] were considered for this work. The population included 41 healthy volunteers with normally functioning TAV and 66 patients diagnosed with at least one of the following conditions: BAV (*n* = 43), dilated AAo (defined as per *z* score > 1.96 [50], *n* = 40) and mild-to-severe AV stenosis (maximum velocity > 2 m/s at the aortic valve, *n* = 20). All subjects had no contraindication to MRI and were free from connective tissue disorders, aortic dissection, coarctation, and other AV diseases. To minimize the presence of potential confounding factors in investigating the influence of AV phenotype on helical flow, patients affected by AAo dilation or AV stenosis were excluded. This resulted in a final cohort of 60 individuals, 41 healthy TAV, and 19 BAV. The BAV phenotype was determined using balanced steady-state free precession cine imaging of the aortic valve.

All participants’ recruitment and research procedures adhered to the principles of the Declaration of Helsinki. The study protocol was approved by the Ethics Committee of the Vall d’Hebron Hospital (Approval No: PR(AG)363/2016) and written informed consent was obtained from all participants.

### MRI Acquisition Protocol and Data Processing

Cardiac MRI was performed on a 1.5-T GE Signa scanner (GE Healthcare, Waukesha, Wisconsin) without the use of intravenous contrast. The imaging protocol included 2D-balanced steady-state free precession cine images and 4D phase contrast MRI (4D flow MRI) with retrospective ECG gating during free breathing. For 4D flow MRI, a radially undersampled acquisition with five-point balanced velocity encoding [51] was employed. Data were acquired across the entire thoracic aorta using the following parameters: field of view 400 × 400 × 400 mm, voxel size 2.5 × 2.5 × 2.5 mm, flip angle 8°, repetition time 4.2–6.4 ms, echo time 1.9–3.7 ms, and reconstructed temporal resolution of 22.3–34.1 ms. Maximum velocity encoding (VENC) was individually adjusted for each participant based on peak aortic valve velocity measured from 2D phase contrast images and ranged from 150 to 200 cm/s. The acquired 4D flow MRI data were corrected for background phase errors related to concomitant gradients, eddy currents, and trajectory deviations of the three-dimensional radial k-space acquisition [51]. Full details of the 4D flow MRI acquisition protocol have been reported in a previous study [52].

**Fig. 1:**
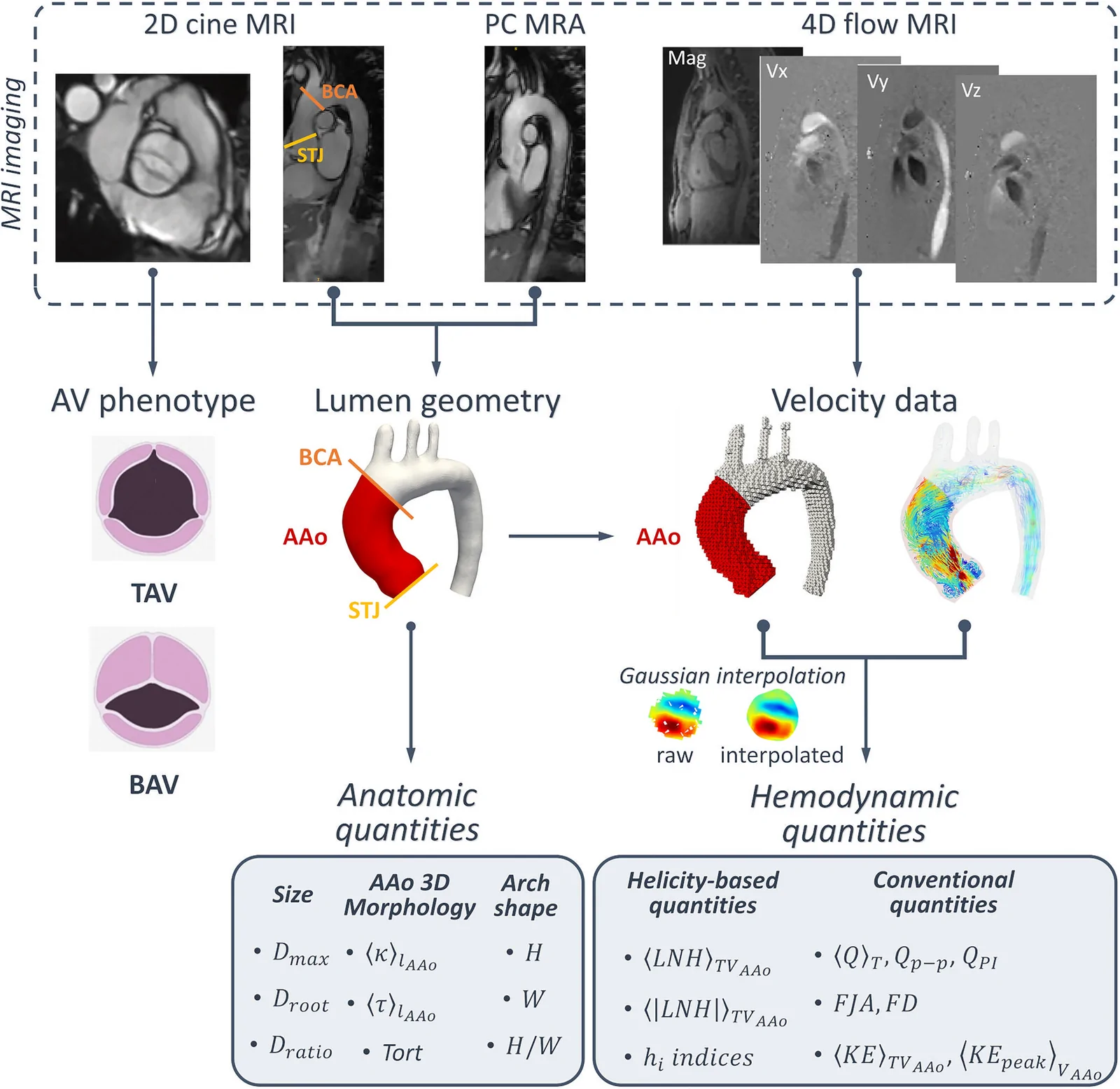
Schematic diagram of the study design, showing how MRI data are used to perform anatomical and hemodynamic characterization in the AAo. *PC MRA* phase contrast MR angiography, *STJ* sinotubular junction, *BCA* brachiocephalic artery, *AAo* ascending aorta, *FJA* flow jet angle, *FD* flow displacement, ${\langle Q\rangle }_{{T}}$ cycle-average aortic blood flow rate, ${Q}_{{p-p}}$ flow rate peak-to-peak amplitude, ${Q}_{{PI}}$ pulsatility index, ${\langle KE\rangle }_{T{V}_{{AAo}}}$ volume- and cycle-average kinetic energy, ${\langle {KE}_{{peak}}\rangle }_{{V}_{{AAo}}}$ volume-average peak kinetic energy, ${D}_{{max}}$ maximum AAo diameter, ${D}_{{root}}$ maximum aortic root diameter, ${D}_{{ratio}}$ diameter ratio ${D}_{{max}}/{D}_{{root}}$, ${\langle \kappa \rangle }_{{l}_{{AAo}}}$ AAo average curvature, ${\langle \tau \rangle }_{{l}_{{AAo}}}$ AAo average torsion, *Tort* AAo tortuosity, H aortic arch height, W aortic arch width

The thoracic aorta was segmented semi-automatically from a 4D flow-derived angiogram. For this study, the AAo region of interest was defined between the sinotubular junction (STJ) and the brachiocephalic trunk, using anatomical landmarks identified on co-registered 2D cine images (Fig. 1). The resulting 3D segmentation of the AAo was then applied as a mask to the acquired 4D flow MRI velocity data for subsequent hemodynamic analysis.

### Hemodynamics Quantitative Characterization

For each subject, aortic phase contrast velocity data were extracted voxel-wise and exported using in-house Matlab code (MathWorks Inc., USA) [52]. The resulting 4D velocity fields were spatially interpolated onto an unstructured mesh (average element size of 1.6 mm) using a Gaussian kernel (kernel size of 3 mm), to increase the accuracy of velocity gradient tensor calculation. The unstructured mesh resolution and the Gaussian kernel size were selected based on a preliminary sensitivity analysis (Supplementary Data). These interpolated datasets served as the basis for computing helicity-based quantities to perform a global characterization of helical flow in the AAo (Table 1) and for computing other conventional hemodynamic quantities (Fig. 1).

**Table 1:** Definition of helicity-based quantities

Helicity-based quantity	Definition	
${\langle LNH\rangle }_{T{V}_{{AAo}}}$	$\frac{1}{T{V}_{AAo}}\int\limits_{T} \int\limits_{V_{AAo}} \frac{{\bf{V}}\cdot {\mathbf \upomega}}{\left|{\bf{V}}\right| \left|{\mathbf \upomega}\right|}dVdt$	$-1\le {\langle LNH\rangle }_{T{V}_{{AAo}}}\le 1$
${\langle \left|LNH\right|\rangle }_{T{V}_{{AAo}}}$	$\frac{1}{T{V}_{{AAo}}}\underset{T}{\overset{\phantom{0}}{\int }}\underset{{V}_{{AAo}}}{\overset{\phantom{0}}{\int }}\left|\frac{\mathbf{V}\cdot {\mathbf \upomega} }{\left|\mathbf{V}\right|\boldsymbol{ }|{\mathbf \upomega}|}\right| dVdt$	$0\le {\langle \left|LNH\right|\rangle }_{T{V}_{{AAo}}}\le 1$
${h}_{1}$	$\frac{1}{T{V}_{{AAo}}}\underset{T}{\overset{\phantom{0}}{\int }}\underset{{V}_{{AAo}}}{\overset{\phantom{0}}{\int }}{\mathbf{V}}\cdot {\mathbf \upomega}\ dVdt$	
${h}_{2}$	$\frac{1}{T{V}_{{AAo}}}\underset{T}{\overset{\phantom{0}}{\int }}\underset{{V}_{{AAo}}}{\overset{\phantom{0}}{\int }}\left|{\mathbf{V}}\cdot{\mathbf \upomega} \right| dVdt$	
${h}_{3}$	$\frac{{h}_{1}}{{h}_{2}}$	$-1\le {h}_{3}\le 1$
${h}_{4}$	$\left|{h}_{3}\right|=\frac{|{h}_{1}|}{{h}_{2}}$	$0\le {h}_{4}\le 1$

$\mathbf{V}$ Blood velocity vector; $\mathbf \upomega$ Blood vorticity vector; ${V}_{{AAo}}$ AAo volume; T temporal observation window

The quantitative analysis of helical flow in this study was based on kinetic helicity density, defined as the internal product between the blood velocity vector ($\mathbf{V}$) and the vorticity vector ($\mathbf \upomega$). The sign of kinetic helicity density indicates the rotational direction of helical flow structures: positive values denote right-handed helical fluid structures (clockwise rotation), whereas negative values denote left-handed helical fluid structures (counterclockwise rotation), both defined relative to the forward flow direction (proximal to distal). To facilitate comparison among subjects and to emphasize topological flow features, kinetic helicity density was locally normalized to velocity and vorticity magnitudes to obtain the local normalized helicity (LNH), a widely adopted quantity for characterizing arterial blood flow [4, 7, 8]. In the present study, the absolute value of the local normalized helicity (|LNH|) was also used to quantify the local alignment between vectors $\mathbf{V}$ and $\mathbf \upomega$. Values of |LNH| close to 1 indicate well-defined helical fluid structures in the AAo, irrespective of rotational direction, whereas |LNH| values approaching 0 indicate an absence of helical flow. Here, the volume- and cycle-average values of LNH and |LNH| were considered, and indicated as ${\langle LNH\rangle }_{T{V}_{{AAo}}}$ and ${\langle \left|LNH\right|\rangle }_{T{V}_{{AAo}}}$, respectively (Table 1). In addition to LNH-related global topological quantities, the AAo helical flow was further characterized in terms of global amount, intensity, and relative rotational direction using other established helicity-based quantities. These quantities were derived by integrating the kinetic helicity density across the AAo fluid domain (volume ${V}_{{AAo}}$) and over an observation window corresponding to an entire cardiac cycle (duration T). Specifically, the following quantities were computed as previously described [7]: cycle-average helicity (${h}_{1}$), helicity intensity (${h}_{2}$), and the signed (${h}_{3}$) and unsigned (${h}_{4}$) helical rotation balance. The quantities ${h}_{1}$ and ${h}_{2}$ measure, respectively, the net amount and the overall intensity of helical flow, averaged over the cardiac cycle. By construction, the helical rotation balance quantities capture the dominance of rotational helical fluid structures. Specifically, ${h}_{3}$ indicates the prevailing rotational direction, with its sign denoting left- or right-handed rotation, while ${h}_{4}$ reflects only the existence of a preferential rotational direction of helical fluid structures. Consequently, ${h}_{1}$, ${h}_{3}$ and ${h}_{4}$ equal 0 in the absence of net (volume- and time-averaged) kinetic helicity density or when reflectional flow symmetry is present. Moreover, ${h}_{3}$ attains a value of − 1 when only left-handed helical flow structures are present in the fluid domain, and + 1 when only right-handed helical flow structures are present.

In addition to helicity-derived quantities, conventional hemodynamic parameters were computed to characterize the main features of AAo blood flow. The aortic blood flow rate waveform, Q(t), measured at the STJ, was used to derive the cycle-average flow rate ${\langle Q\rangle }_{{T}}$, the peak-to-peak amplitude ${Q}_{{p-p}}$, and the pulsatility index ${Q}_{{PI}}$, as described in previous studies [35, 53]. The eccentricity of the AV systolic outflow jet was quantified at the STJ cross-section in terms of flow jet angle (FJA) and normalized flow displacement (FD) [54], the latter measuring the distance between the anatomical centroid of the aorta and the centroid of the forward velocity profile, normalized to the luminal diameter. Finally, the kinetic energy (KE) associated with the large-scale AAo blood flow was quantified in terms of volume- and cycle-average KE (${\langle KE\rangle }_{T{V}_{{AAo}}}$) and volume average peak KE (${\langle {KE}_{{peak}}\rangle }_{{V}_{{AAo}}}$) [35, 53].

### Anatomical Quantitative Characterization

The main aortic anatomical features were assessed in terms of AAo aortic size, 3D morphology, and aortic arch shape (Fig. 1), given their well-established influence on aortic intravascular hemodynamics [35, 55, 56]. Aortic size was quantified on appropriate cine images using the maximum AAo diameter (${D}_{{max}}$), a key diagnostic marker of aortic dilation [57], the maximum aortic root diameter (${D}_{{root}}$), and their ratio (${D}_{{ratio}}= {D}_{{max}}/{D}_{{root}}$) as an indicator of relative AAo dilation [58]. A robust centerline-based approach was used to characterize aortic morphology, quantifying the average curvature (${\langle \kappa \rangle }_{{l}_{{AAo}}}$) and torsion (${\langle \tau \rangle }_{{l}_{{AAo}}}$) along the AAo centerline’s length (${l}_{{AAo}}$), and AAo tortuosity (*Tort*), following previously proposed approaches [20, 59]. The aortic arch shape was also characterized using a centerline-based approach, computing arch height (H) and width (W), and their ratio (H/W) [35].

### Statistical Analysis

All variables are reported as median values and interquartile range (IQR). Group differences in helicity-based quantities between TAV and BAV subjects were assessed using the Mann–Whitney *U* test. Bivariate associations among helicity-based quantities, as well as between helicity-based quantities and anatomical or conventional hemodynamic parameters, were evaluated using partial Spearman rank correlation coefficients (R) adjusted for demographic and clinical variables when appropriate [60]. To identify anatomical and hemodynamic features independently associated with the AV phenotype, univariate and bivariate logistic generalized linear regression models were employed, with BAV as the categorical response variable and anatomical, conventional hemodynamic parameters, and helicity-based quantities as independent regressors. Odds ratios (OR) per unit of standard deviation increase in each independent variable, along with their 95% confidence intervals (95% CI), were derived from the exponential of the standardized regression coefficients. Model goodness of fit was evaluated using the adjusted coefficient of variation ($\mathrm{R}_{\mathrm{adj}}^{2}$) and log-likelihood (*logL*) values. Statistical significance was set at a *p* < 0.05. All the statistical analysis was conducted within the MATLAB environment (MathWorks Inc., USA).

## Results

Descriptive statistics for the demographic, clinical, helicity-based, conventional hemodynamic and anatomical parameters of the study population are presented in Table 2. Compared with TAV subjects, BAV patients exhibited significantly larger AAo (in terms of ${D}_{{max}}$ and ${D}_{{root}}$), greater AV flow eccentricity (as measured by FJA and FD), and higher flow rate pulsatility (${Q}_{{PI}}$).

**Table 2:** Demographic, clinical, anatomical and conventional hemodynamic data of the TAV and BAV cohorts under study

	TAV	BAV	*p*		TAV	BAV	*p*
Demographic and clinical quantities				Helicity-based quantities			
N°	41	19		${\langle LNH\rangle }_{T{V}_{{AAo}}}$[−]	0.007 (− 0.010–0.017)	0.008 (− 0.013–0.036)	–
Age [years]	36.0 (30.0–46.2)	46.0 (40.3–55.4)	●	${\langle \left|LNH\right|\rangle }_{T{V}_{{AAo}}}$[−]	0.451 (0.447–0.456)	0.436 (0.430–0.439)	★
Sex, female [N, (%)]	15 (36.6)	6 (31.6)	–	${h}_{1}$[m s^−2^]	− 0.049 (− 0.287–0.215)	0.040 (− 0.272–0.481)	–
BSA [m^2^]	1.82 (1.73-1.94)	1.83 (1.66-1.93)	–	${h}_{2}$[m s^−2^]	7.51 (5.44-9.57)	4.05 (3.46-5.35)	★
SBP [mmHg]	120.5 (115.0–129.5)	128.0 (116.5-142.5)	–	${h}_{3}$[−]	− 0.007 (− 0.059–0.034)	0.008 (− 0.094–0.097)	–
DBP [mmHg]	70.0 (61.5-76.5)	75.0 (69.7-81.0)	–	${h}_{4}$[−]	0.043 (0.014–0.090)	0.097 (0.047–0.137)	●
Conventional hemodynamic quantities				Anatomic quantities			
FJA[°]	8.96 (6.17-11.99)	14.5 (9.55-21.80)	★	${D}_{{max}}$[mm]	30.7 (26.2-32.9)	36.7 (33.2-39.7)	★
FD[−]	0.07 (0.05–0.09)	0.14 (0.08–0.19)	★	${D}_{{root}}$[mm]	30.0 (27.0–33.2)	33.0 (31.2-35.7)	▲
${\langle KE\rangle }_{T{V}_{{AAo}}}$[Pa]	46.69 (35.2-56.0)	36.12 (28.4-47.1)	–	${D}_{{ratio}}$[−]	1.01 (0.87-1.09)	1.03 (0.97-1.14)	–
${\langle {KE}_{{peak}}\rangle }_{{V}_{{AAo}}}$[Pa]	170.3 (130.6-268.2)	156.0 (106.9-205.6)	–	${\langle \kappa \rangle }_{{l}_{{AAo}}}$[mm^−1^]	0.029 (0.026–0.030)	0.028 (0.025–0.032)	–
${\langle Q\rangle }_{{T}}$[l min^−1^]	3.74 (3.06-4.25)	3.87 (2.53-4.50)	–	${\langle \tau \rangle }_{{l}_{{AAo}}}$[mm^−1^]	0.110 (0.088–0.170)	0.121 (0.108–0.163)	–
${Q}_{{p-p}}$[l min^−1^]	19.0 (15.7-24.8)	23.2 (18.6-26.5)	–	H[mm]	27.8 (24.1-32.8)	32.5 (29.8-34.9)	●
${Q}_{{PI}}$[−]	5.33 (4.64-6.16)	6.10 (5.79-7.62)	▲	W[mm]	64.4 (57.2-70.7)	71.8 (64.5-78.4)	●
				H/W[−]	0.43 (0.38–0.52)	0.44 (0.40–0.50)	–
				Tort[−]	18.1 (15.3-22.5)	18.1 (12.9-28.4)	–

BSA was computed using the Du Bois equation [61]. Data are presented as median value (interquartile range, IQR) or number (percentage). Differences between TAV and BAV subjects were assessed using Mann–Whitney *U* tests

*N°* number of subjects in the cohort, *BSA* body surface area, *SBP* systolic blood pressure, *DBP* diastolic blood pressure

Statistical significance is indicated with ● * p* < 0.05; ▲ * p* < 0.01; ★ *p* < 0.001; – non-significant

**Fig. 2:**
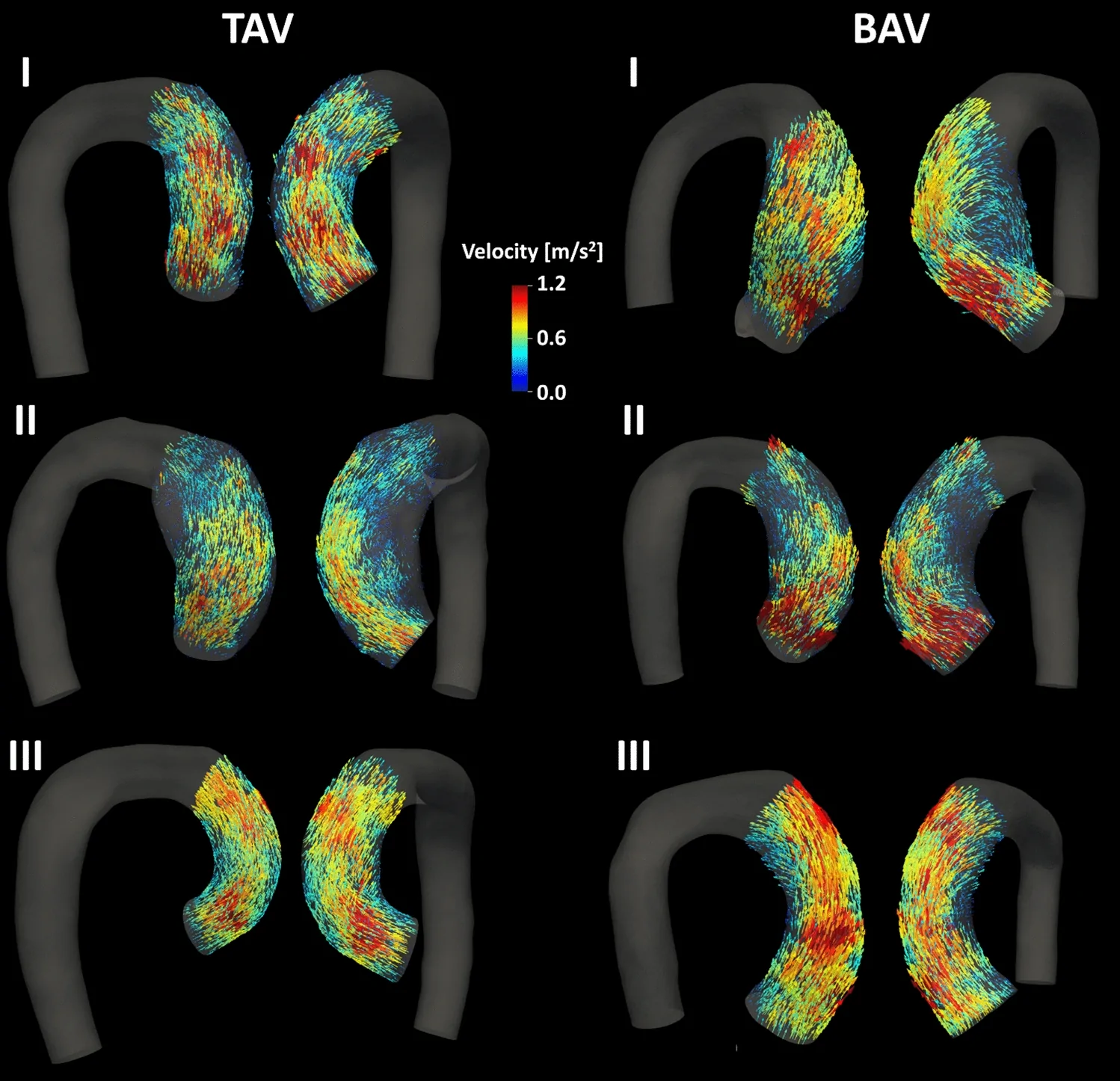
Visualization of blood flow vector fields in the AAo (posterior and right-anterior view) at around peak systole in six representative subjects (three TAV and three BAV). The selected subjects were sampled from each tertile of ${\langle \left|LNH\right|\rangle }_{T{V}_{{AAo}}}$. Colors represent velocity magnitude. Reconstructed geometries are shown with different scales

A qualitative overview of AAo hemodynamics is shown in Fig. 2, which depicts blood flow vector fields at around peak systole from six representative subjects (three TAV and three BAV). In TAV individuals, systolic outflow at the STJ typically appears as a uniform, centrally directed jet (TAV subjects I and III in Fig. 2). In contrast, BAV patients often exhibit an eccentric, high-velocity jet directed toward the AAo outer wall, accompanied by a prominent low-velocity recirculation zone along the inner wall (BAV patients I and II in Fig. 2).

**Fig. 3:**
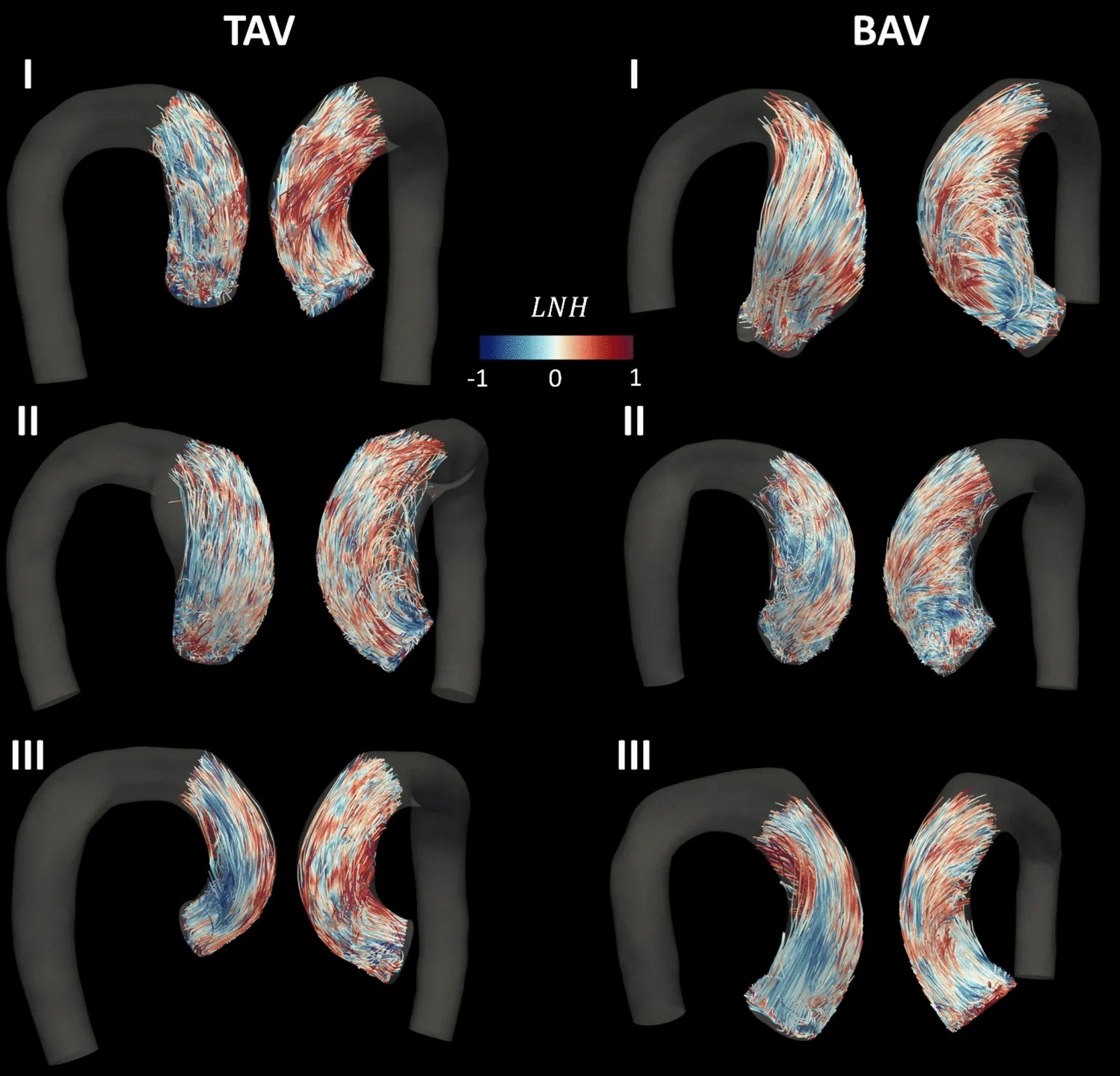
Pathline visualization of aortic helical flow (posterior and right-anterior view) during systolic deceleration in six representative subjects (three TAV and three BAV). The selected subjects were sampled from each tertile of ${\langle \left|LNH\right|\rangle }_{T{V}_{{AAo}}}$. Virtual particles were released in the STJ at peak systole and color coded with local instantaneous *LNH* values. Reconstructed geometries are shown with different scales

To illustrate the helical-shaped organization of AAo blood flow, Fig. 3 shows 3D pathlines color-coded with local instantaneous LNH values for the same representative subjects depicted in Fig. 2. These visualizations reveal coherent, counter-rotating helical flow structures in the AAo for both TAV and BAV subjects. Regardless of AV phenotype, right-handed helical fluid structures (positive LNH) consistently appear near the AAo inner wall (right views in Fig. 3) and extend distally along the arch, whereas left-handed helical fluid structures (negative LNH) are localized along the outer wall (left views in Fig. 3). Qualitatively, coherent helical flow appears more prominent in subjects with more eccentric systolic outflow jet—particularly TAV subject II and BAV patients I and II in Figs. 2 and 3—suggesting a direct link between jet eccentricity and the propensity of the flow field to form coherent helical patterns.

### Impact of AV Phenotype on AAo Helicity

**Fig. 4:**
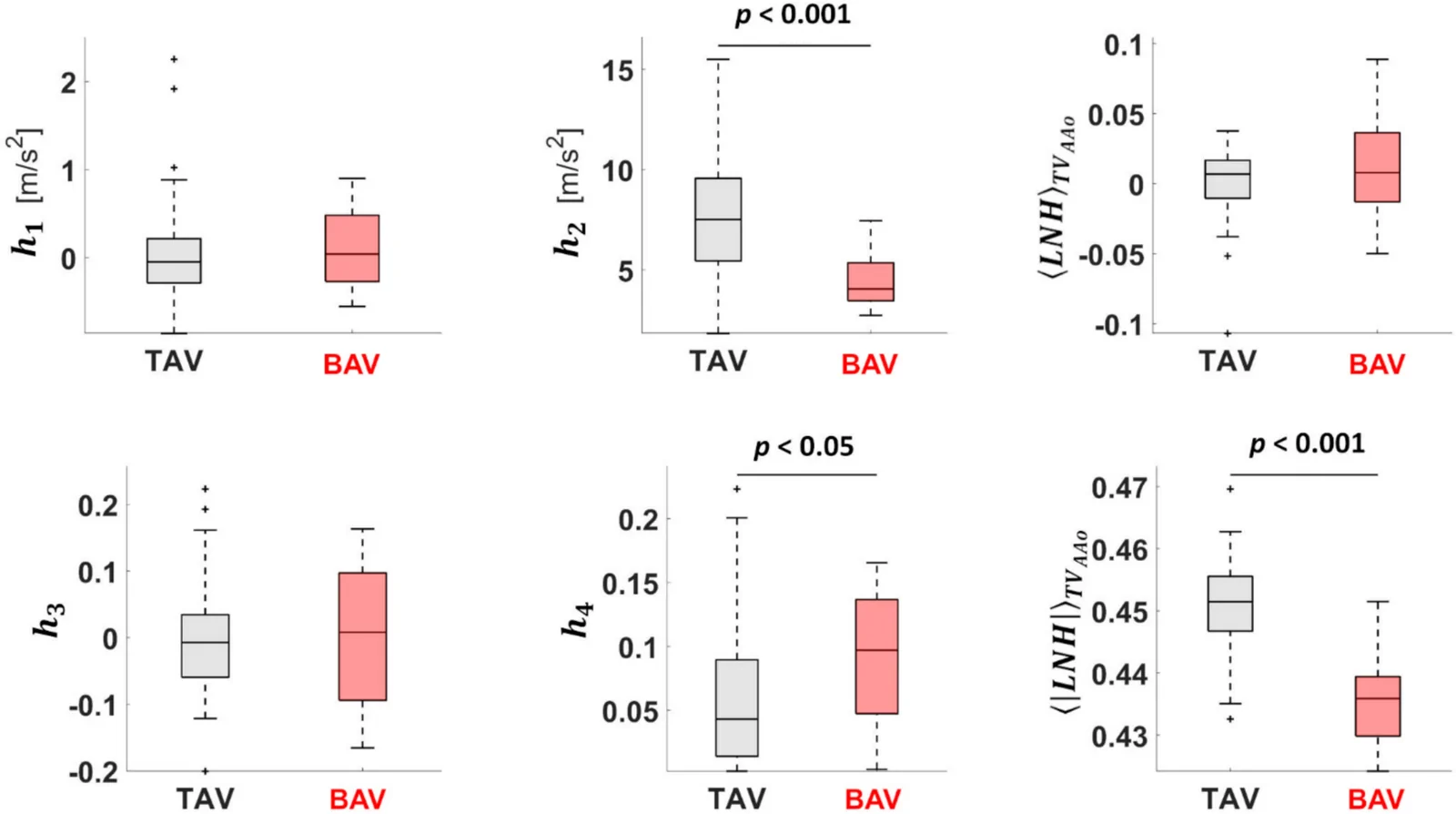
Boxplots comparing helicity-based quantities in the TAV and BAV groups. In the plots, the median is indicated by the black line, and the box indicates the interquartile range (IQR). Differences between the two groups of TAV and BAV subjects were assessed using Mann–Whitney *U* tests

To quantitatively assess helical flow in AAo, Fig. 4 presents boxplots of helicity-based quantities for the TAV and BAV cohorts. These quantities were computed as described in Table 1, using the entire cardiac cycle as the observation window. The corresponding boxplots obtained by analyzing separately systole and diastole as observation windows are provided in Fig. S1 of the Supplementary Data. Statistical analysis revealed that both ${h}_{2}$ and ${\langle \left|LNH\right|\rangle }_{T{V}_{{AAo}}}$ were significantly higher in TAV subjects (*p* < 0.001), indicating greater helicity intensity and a stronger propensity for the aortic flow to form organized helical fluid structures in individuals with a normal AV phenotype. In contrast, ${h}_{4}$ was significantly higher in BAV patients (*p* < 0.05), suggesting that BAV phenotype is associated with more unbalanced counter-rotating helical fluid structures compared with TAV. Nonetheless, the overall low ${h}_{4}$ values observed in both groups (Table 2) indicate that, on average, counter-rotating helical flow patterns remained largely balanced.

Bivariate correlations with demographic and clinical variables revealed distinct patterns across groups. In TAV subjects, ${\langle \left|LNH\right|\rangle }_{T{V}_{{AAo}}}$ was larger in men (R = − 0.39, *p* < 0.05) and it was positively correlated with age (R = 0.34, *p* < 0.05) and BSA (R = 0.41, *p* < 0.05). In BAV patients, both ${h}_{1}$ and ${h}_{3}$ were inversely correlated with age (R = − 0.49 and R = − 0.47, *p* < 0.05, respectively), ${h}_{4}$ was larger in women (R = 0.50, *p* < 0.05) and inversely correlated with BSA (R = − 0.56, *p* < 0.05), while ${\langle LNH\rangle }_{T{V}_{{AAo}}}$ showed a positive correlation with diastolic blood pressure (DBP, R = 0.49, *p* < 0.05). When considering the entire population (i.e., of TAV and BAV together), ${h}_{1}$, ${h}_{2}$, and ${h}_{3}$ were inversely correlated with age (R = − 0.27,* p* < 0.05; R = − 0.41 and –0.38, *p* < 0.01, respectively), and ${h}_{4}$ was positively correlated with systolic blood pressure (SBP, R = 0.34, *p* < 0.05).

**Fig. 5:**
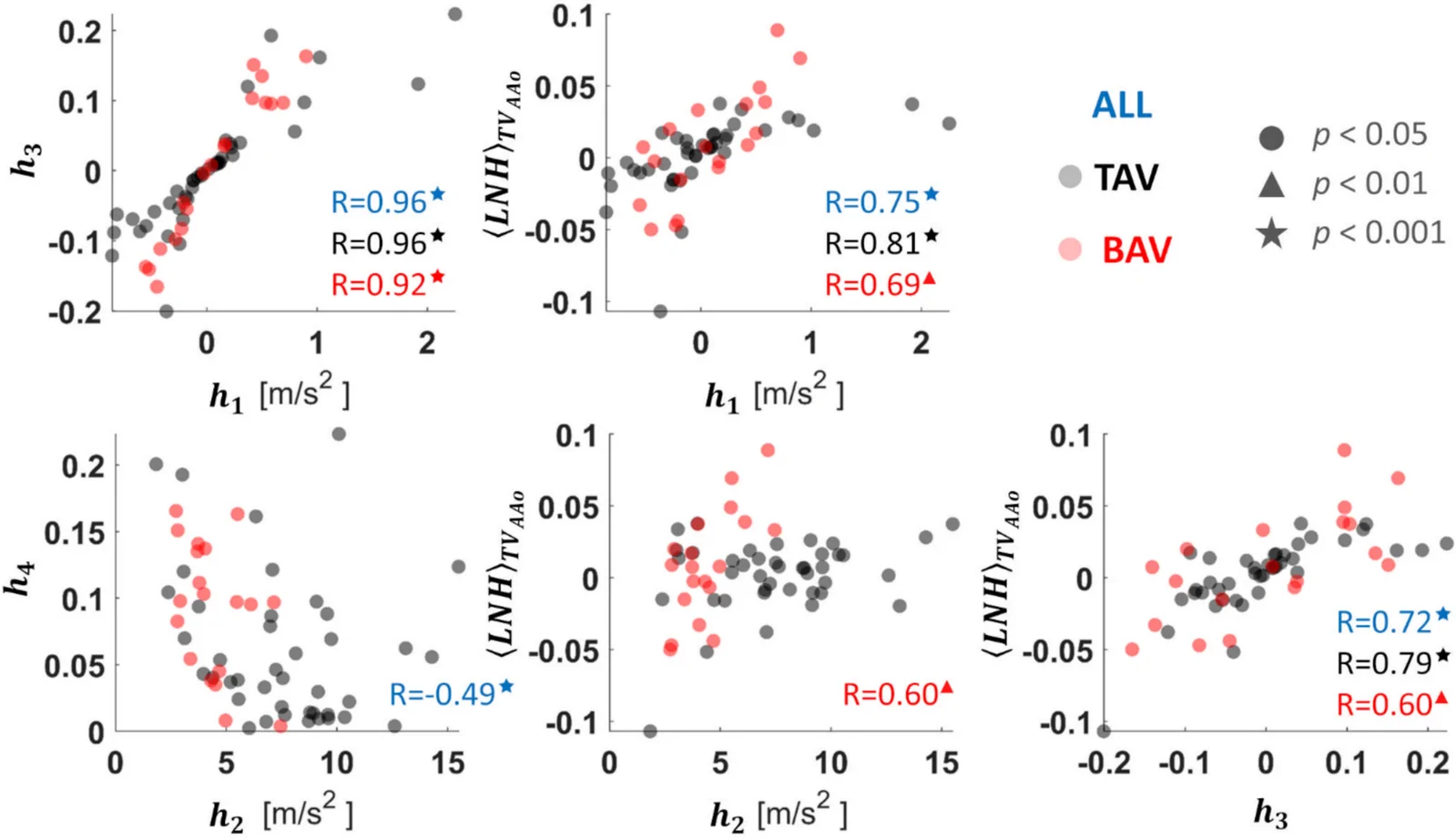
Scatter plots showing the significant associations among helicity-based quantities in the entire population (ALL), and in the TAV and BAV groups. The corresponding adjusted partial Spearman correlation coefficients R are also reported. Statistical significance is indicated with ● *p* < 0.05; ▲ *p* < 0.01; ★ *p* < 0.001

Associations among helicity-based quantities across the study population were examined using partial Spearman correlations, adjusted for demographic and clinical variables when appropriate. As illustrated in Fig. 5, ${h}_{1}$ and ${h}_{3}$ were strongly positively correlated—as expected, given the definition of ${h}_{3}$ (see Table 1)—in both the TAV and BAV cohorts, as well as in the combined population (ALL), with correlation coefficients ranging from R = 0.92 to R = 0.96 (*p* < 0.001). Moderate-to-strong correlations also emerged between ${\langle LNH\rangle }_{T{V}_{{AAo}}}$ and both ${h}_{1}$ (R = 0.69–0.81, *p* < 0.01) and ${h}_{3}$ (R = 0.60–0.79, *p* < 0.01), consistently across AV phenotypes. Additionally, ${h}_{2}$ showed a positive correlation with ${\langle LNH\rangle }_{T{V}_{{AAo}}}$ in the BAV cohort (R = 0.60, *p* < 0.01) and an inverse correlation with ${h}_{4}$ in the combined population (R = − 0.49, *p* < 0.001).

### Hemodynamic and Anatomical Determinants of AAo Helicity

**Fig. 6:**
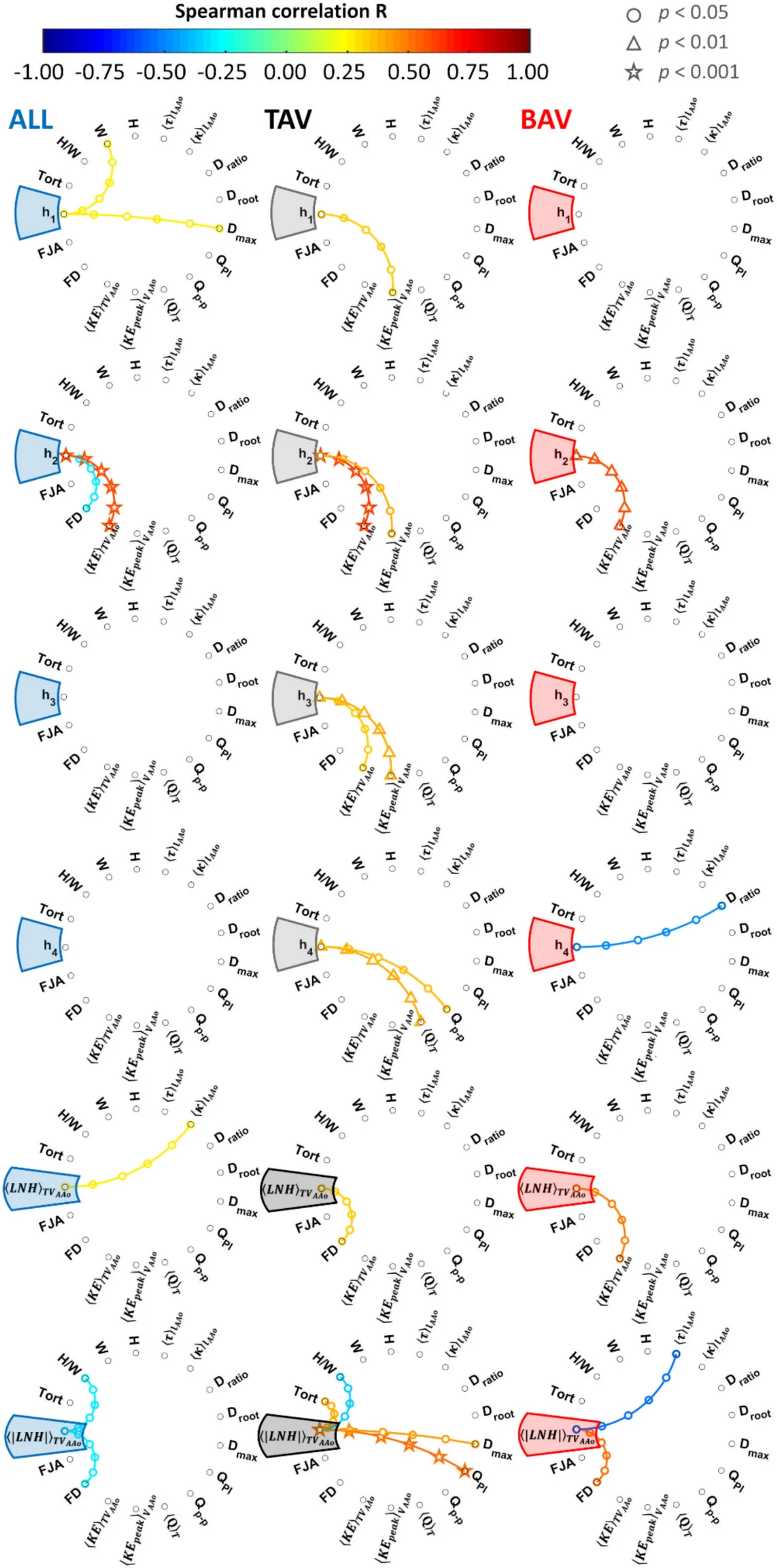
Circular correlation plots showing the significant associations between helicity-based quantities and anatomical or conventional hemodynamic quantities in the entire population (ALL), and in the TAV and BAV groups. In the plots, colors represent the corresponding adjusted partial Spearman correlation coefficients R. Statistical significance is indicated with: ● *p* < 0.05; ▲ *p* < 0.01; ★ *p* < 0.001

To further explore the determinants of AAo helical flow, adjusted partial Spearman correlations were computed between helicity-based quantities and anatomical or conventional hemodynamic quantities. All significant associations are summarized in the circular correlation plots shown in Fig. 6, reported separately for the combined population and for the TAV and BAV cohorts. As expected from its definition, ${h}_{2}$ showed a positive correlation with the AAo cycle-average kinetic energy ${\langle KE\rangle }_{T{V}_{{AAo}}}$ in the combined population (R = 0.59, *p* < 0.001) as well as within the TAV (R = 0.63, *p* < 0.001) and BAV (R = 0.60, *p* < 0.01) subgroups. On the contrary, ${h}_{2}$ showed significant negative correlation with systolic outflow jet flow displacement (FD) (R = − 0.31, *p* < 0.05) in the combined population.

Notably, in BAV patients, higher ${\langle \left|LNH\right|\rangle }_{T{V}_{{AAo}}}$ values—reflecting a stronger propensity for helical flow organization—were positively correlated with FD (R = 0.54, *p* < 0.05) and negatively with aortic torsion (R = − 0.56, *p* < 0.05). In TAV subjects, ${\langle \left|LNH\right|\rangle }_{T{V}_{{AAo}}}$ was instead associated with different determinants, showing positive correlations with ${Q}_{{PI}}$ (R = 0.56, *p* < 0.001), ${D}_{{max}}$ (R = 0.40, *p* < 0.05) and *Tort* (R = 0.39, *p* < 0.05). Additionally, ${h}_{4}$ exhibited mild positive correlation with ${\langle Q\rangle }_{{T}}$ (R = 0.40, *p* < 0.01) and ${Q}_{{p-p}}$ (R = 0.36, *p* < 0.05) in TAV subjects, whereas in BAV patients it was inversely associated with ${D}_{{ratio}}$ (R = − 0.48, *p* < 0.05).

### Logistic Regression Models of BAV Phenotype

Results from the univariate and bivariate logistic regression analyses linking the BAV phenotype with anatomical, hemodynamic and helical flow features are summarized in Table 3, which highlights the top-ranked three statistical models in each category. Among single-feature models, the strongest association with the BAV phenotype was observed for ${\langle \left|LNH\right|\rangle }_{T{V}_{{AAo}}}$ (${\mathrm{R}}_{\mathrm{adj}}^{2}$ = 0.515), followed by FD (${\mathrm{R}}_{\mathrm{adj}}^{2}$ = 0.433) and ${D}_{{max}}$ (${\mathrm{R}}_{\mathrm{adj}}^{2}$ = 0.298). When extending the analysis to bivariate models, the BAV phenotype showed the strongest associations when ${\langle \left|LNH\right|\rangle }_{T{V}_{{AAo}}}$ was combined with another anatomical or conventional hemodynamic quantity. The combination of ${\langle \left|LNH\right|\rangle }_{T{V}_{{AAo}}}$ and aortic arch width W achieved the highest explanatory power (${\mathrm{R}}_{\mathrm{adj}}^{2}$ = 0.881), followed by ${\langle \left|LNH\right|\rangle }_{T{V}_{{AAo}}}$ with ${D}_{{max}}$ (${\mathrm{R}}_{\mathrm{adj}}^{2}$ = 0.857), and ${\langle \left|LNH\right|\rangle }_{T{V}_{{AAo}}}$ with FD (${\mathrm{R}}_{\mathrm{adj}}^{2}$ = 0.807). These findings underscore the pivotal role of the bicuspid aortic valve phenotype in shaping helical flow topology, especially when considered alongside anatomical or conventional hemodynamic quantities.

**Table 3:** Results from logistic regression analyses highlighting the association of anatomical, conventional hemodynamic and helicity-based quantities with the categorical response variable BAV

	Univariate models BAV ~* X*_1_			Bivariate models BAV ~ * X*_1_ + * X*_2_		
N.	1.	2.	3.	1.	2.	3.
*X*_1_	${\langle \left|LNH\right|\rangle }_{T{V}_{{AAo}}}$	FD	${D}_{{max}}$	${\langle \left|LNH\right|\rangle }_{T{V}_{{AAo}}}$	${\langle \left|LNH\right|\rangle }_{T{V}_{{AAo}}}$	${\langle \left|LNH\right|\rangle }_{T{V}_{{AAo}}}$
*p* value	< 0.0001	< 0.001	< 0.001	< 0.01	< 0.01	< 0.01
OR (95% LCI to UCI)	0.0999 (0.0314 to 0.3181)	5.7514 (2.1600 to 15.3139)	4.2068 (1.8012 to 9.8251)	0.0100 (4.15e^−04^ to 0.2432)	0.0110 (5.31e^−04^ to 0.2273)	0.0699 (0.012 to 0.4081)
*X*_2_	–	–	–	W	${D}_{{max}}$	FD
*p* value	–	–	–	< 0.05	< 0.01	< 0.05
OR (95% LCI to UCI)	–	–	–	15.3855 (1.6323 to 145.0229)	8.4579 (1.7551 to 40.7599)	4.8903 (1.3386 to 17.8656)
$\mathrm{R}_{\mathrm{adj}}^{2}$	0.515	0.433	0.298	0.881	0.857	0.807
*logL*	− 19.786	− 24.976	− 27.961	− 5.008	− 6.051	− 7.239

The top three univariate and bivariate regression models are reported, ranked by model goodness-of-fit in terms of the adjusted coefficient of variation $\mathrm{R}_{\mathrm{adj}}^{2}$. * X*_i_ = independent regressor; OR = odds ratio of the univariate and bivariate analyses; 95% LCI to UCI = 95% lower and upper confidence intervals; *logL* = log-likelihood function of the univariate and bivariate models

## Discussion

The physiological significance of helical flow in the human cardiovascular system—particularly in the aorta—has become increasingly recognized. Both experimental and computational studies have demonstrated that helical flow patterns support vascular health by suppressing flow disturbances, reducing flow separation, stagnation, and recirculation, thereby reducing the pro-atherogenic action of WSS and ensuring adequate mass transport [8, 10, 18]. The advent of 4D flow MRI—now considered the gold standard, non-invasive method for assessing aortic hemodynamics—has made it possible to characterize helical flow in vivo. This is especially valuable in the study of BAV disease, due to its high prevalence and strong association with AAo dilation.

Previous studies have described in vivo abnormal AAo hemodynamics in patients with BAV, often reporting overly pronounced, supraphysiological helical flow patterns [40, 44, 62]. However, these characterizations relied on visual assessments of rotational flow around the vessel centerline, therefore being mostly qualitative and observer-dependent, [26, 33, 36, 40, 62, 63]. In several cases, qualitative evaluation was based on instantaneous velocity streamlines [26, 40, 63] rather than on time-resolved 3D pathlines. The latter provide a more accurate depiction of blood particle trajectories within a dynamic velocity field, thus enabling a theoretically sounder assessment of helicity-related quantities [22, 23].

To address these limitations, distinct approaches were previously introduced to depict aortic helical flow from 4D flow MRI acquisitions; however, several of these approaches mainly focused either on 2D analysis planes [29] or instantaneous snapshots of the cardiac cycle [28] or on partial contributions to helical flow based only on the vorticity field [44–47]. In the attempt to bridge this gap, in the present study we performed a comprehensive quantitative, theoretically-grounded characterization of the intensity and topology of aortic helical flow from 4D flow MRI in vivo data, by adopting a fluid mechanics-based approach identifying helical fluid structures as linked and knotted vortex lines [49]. Our aim was to investigate how AV phenotype shapes AAo helical flow patterns, and to identify the key anatomical and hemodynamic determinants of helical flow in subjects with TAV and BAV. Accordingly, the reported findings apply specifically to subjects with clinically non-dilated ascending aortas and with non-stenotic valves, therefore excluding AAo dilation and AV stenosis which are both major mediators of aortic flow patterns and could mask the real impact of valve phenotype on helical flow.

### Impact of AV Phenotype on AAo Helical Flow

Our analysis shows that the BAV phenotype significantly reduces both helicity intensity (quantified by ${h}_{2}$) and the predisposition of blood flow to form coherent helical patterns (quantified by ${\langle \left|LNH\right|\rangle }_{T{V}_{{AAo}}}$). Pathline analysis during systolic deceleration revealed two coherent, counter-rotating helical fluid structures in both TAV and BAV subjects. Specifically, a dominant right-handed helical flow pattern formed near the inner wall, while a left-handed one formed near the outer wall (Fig. 3). This bihelical flow arrangement in the AAo can be interpreted as the result of axial (through-plane) transport—responsible for downstream perfusion—combined with in-plane, Dean-like secondary flow patterns driven primarily by local AAo curvature [12–14].

The reduction in ${\langle \left|LNH\right|\rangle }_{T{V}_{{AAo}}}$ observed in the BAV cohort (Fig. 4) contrasts with findings from Garcia et al. [28], who reported higher local normalized helicity in BAV patients when analyzing AAo regions exceeding a predefined instantaneous |LNH| threshold value at selected cardiac phases. This discrepancy may partly reflect differences in cohort characteristics: in Garcia et al., BAV patients had significantly larger AAo diameters than their TAV counterparts, potentially introducing an AAo size-related bias. Moreover, their BAV cohort largely included patients with concomitant AV stenosis and/or regurgitation—conditions known to independently impact on helical flow—which were excluded from our study to minimize confounding effects. Lorenz et al. [29] also reported higher helicity in BAV compared with healthy controls, although their analysis was limited to 2D aortic cross-sections and based on a relatively small cohort (12 TAV and four BAV with non-dilated AAo and without concomitant AV disease).

In this first in vivo study quantitatively comparing global AAo helicity intensity (${h}_{2}$) between TAV and BAV phenotypes, we show that the altered hemodynamics associated with BAV markedly reduces helicity intensity (Fig. 4). This finding is consistent with a previous study [31] linking aberrant flow in degenerative ascending thoracic aortic aneurysms to reduced normalized $|\mathbf{V}\cdot {\boldsymbol{\upomega}}|$—a quantity directly related to ${h}_{2}$—which may contribute to heterogeneous wall shear stress distributions, and subsequent wall remodeling. The disruptive impact of BAV phenotype on helicity intensity also supports the well-established role of physiological helical flow in maintaining healthy aortic hemodynamics [8, 10, 11, 13, 14]. Notably, when helicity intensity was evaluated locally within a small region distal to the AV—rather than over the entire AAo volume as in Table 1—local ${h}_{2}$ values were higher than the corresponding global values, as expected given the locally elevated velocities and its gradients generated by the aortic jet. Nevertheless, TAV subjects still exhibited higher local ${h}_{2}$ than BAV patients. This indicates that the impact of valve phenotype on helicity intensity is not solely attributable to a localized jet-driven mechanism, but is consistent with a sustained downstream reorganization of blood flow throughout the AAo.

### Main Determinants of AAo Helical Flow in TAV and BAV Phenotypes

Bivariate analyses with demographic variables revealed a negative association between age and ${h}_{1}$ and ${h}_{3}$ in BAV patients, and between age and ${h}_{2}$ in the combined population. These findings are consistent with prior studies reporting that helical flow patterns in the thoracic aorta become less prevalent with increasing age [56, 64], and that non-normalized helicity quantities—conceptually related to ${h}_{1}$ and ${h}_{2}$—tend to decrease over time [65, 66]. This age dependence is plausibly attributable to established age-related changes in aortic structure and hemodynamics, including increased stiffness, progressive dilation, and geometric remodeling, all of which can alter helical flow organization.

Correlation analyses with anatomical and conventional hemodynamic quantities revealed distinct determinants of AAo helical flow in TAV and BAV phenotypes. In healthy TAV individuals, flow rate pulsatility (${Q}_{{PI}}$) emerged as the primary determinant of AAo helical flow topology, showing a positive correlation with ${\langle \left|LNH\right|\rangle }_{T{V}_{{AAo}}}$ (R = 0.56, *p* < 0.001, Fig. 6). This finding supports early in vitro evidence that pulsatility, in combination with aortic arch curvature, promotes helical flow [12], and is consistent with numerical simulations reporting higher kinetic helicity density under pulsatile versus steady flow in the thoracic aorta [67].

Analyzing anatomical factors, ${\langle \left|LNH\right|\rangle }_{T{V}_{{AAo}}}$ showed a positive correlation with maximum aortic diameter (${D}_{{max}}$) in TAV subjects (R = 0.40, *p* < 0.05, Fig. 6). This aligns with findings from Dyverfeldt et al. [20], who reported a moderate positive correlation between |LNH| near peak systole and mean aortic diameter. In the same study, systolic |LNH| was also significantly higher in older individuals, consistent with our observation of a positive—though weaker—correlation between ${\langle \left|LNH\right|\rangle }_{T{V}_{{AAo}}}$ and age in TAV subjects (R = 0.34, *p* < 0.05). Similarly to Dyverfeldt et al. [20], here a significant but moderate correlation emerged between helical flow topology and aortic tortuosity in TAV subjects (*Tort vs.* ${\langle \left|LNH\right|\rangle }_{T{V}_{{AAo}}}$: R = 0.39, *p* < 0.05, Fig. 6). As suggested in [20], the age-related increase in |LNH| observed in TAV subjects may be associated with progressive aortic tortuosity, which can in turn be attributed, at least in part, to progressive increase in blood pressure and elastin degradation.

In BAV patients, normalized flow displacement (FD) emerged as the primary determinant of helical flow topology. The positive association between FD and the propensity to form coherent helical flow patterns (${\langle \left|LNH\right|\rangle }_{T{V}_{{AAo}}}$ *vs.* FD: R = 0.54, *p* < 0.05, Fig. 6) suggests that BAV-related flow eccentricity may promote helical flow distal to the AV, potentially acting as a compensatory mechanism that delays transition to turbulence. This interpretation (i) is consistent with experimental observations of lower velocity fluctuations and reduced turbulent kinetic energy in eccentric BAV jet configurations compared with central jets [68], and (ii) is supported by the flow-stabilizing role of helicity, which can partially inhibit the energy cascade from large to small scales [19, 69]. Our findings also agree with prior studies linking the eccentric BAV jet to the presence of pronounced coherent rotational fluid structures in the aortic flow field [26, 40, 70, 71]. Moreover, specific BAV fusion types and the presence of AV stenosis have been reported to further accentuate flow displacement and strengthen secondary flow components [36, 44, 45, 72, 73].

Notably, although FD promotes coherent helical fluid structures (higher ${\langle \left|LNH\right|\rangle }_{T{V}_{{AAo}}}$), this was not accompanied by an increase in helicity intensity (${h}_{2}$). A plausible explanation is that, while ${h}_{2}$ correlates with average kinetic energy (${\langle KE\rangle }_{T{V}_{{AAo}}}$), eccentric jets in BAV generate large recirculation zones with low velocity in the AAo. This lowers KE thereby hindering a corresponding rise in helicity intensity. This is also supported by the negative correlation emerged between FD and ${h}_{2}$ in the combined population (R = − 0.31, *p* < 0.05, Fig. 6). Such a reduction in intensity, despite preserved flow topology, may weaken the protective role of helicity and help explain the pathogenic contribution of highly rotational, eccentric flows to AAo dilation in BAV subjects [34, 44, 52]. In addition, eccentric and helical flow in BAV individuals have been consistently associated with asymmetric WSS distributions in the AAo, a factor implicated in vascular degeneration [45, 71, 74–76].

### Helical Flow Topology as a Hallmark of BAV Phenotype

Univariate regression analyses identified helical flow topology as the strongest discriminator between TAV and BAV phenotypes. In detail, ${\langle \left|LNH\right|\rangle }_{T{V}_{{AAo}}}$ proved highly sensitive to the flow disorganization introduced by the bicuspid anatomy, showing greater explanatory power (${\mathrm{R}}_{\mathrm{adj}}^{2}$ = 0.515, Table 3) than FD. Although FD has previously been established as a robust marker of valve phenotype [34, 36, 45, 77], its association in our cohort was weaker than that of ${\langle \left|LNH\right|\rangle }_{T{V}_{{AAo}}}$ (${\mathrm{R}}_{\mathrm{adj}}^{2}$ = 0.433, Table 3). Bivariate models further reinforced the discriminative role of ${\langle \left|LNH\right|\rangle }_{T{V}_{{AAo}}}$, with explanatory power increasing substantially (up to ${\mathrm{R}}_{\mathrm{adj}}^{2}$ = 0.881) when combined with anatomical (arch width W or maximum AAo diameter ${D}_{{max}}$) or conventional hemodynamic parameters (FD). Together, these findings suggest that integrating anatomical and hemodynamic features with helical flow topology yields a powerful, in vivo quantifiable morpho-hemodynamic signature of BAV, with significant promise for both diagnostic and prognostic applications in BAV individuals’ management.

### Limitations of the Study

This study has several limitations. First, computation of helicity quantities might be influenced by the spatial resolution of the raw data and be sensitive to 4D flow MRI velocity encoding. Nonetheless, because all subjects were analyzed with the same acquisition and processing methods, group comparisons remain substantially unbiased. Although a formal reproducibility assessment was not performed in the present study, prior work has reported good inter- and intra-observer agreement for 4D flow-derived velocity fields and aortic hemodynamic metrics [28, 29, 78–80], as well as good-to-excellent scan–rescan reproducibility [81]. In addition, all data were acquired on a single scanner using a standardized acquisition protocol, thereby minimizing inter-scanner variability within the dataset. Second, the finite spatial resolution of clinical 4D flow MRI may limit the accuracy of velocity gradient estimation. To mitigate this limitation, we interpolated the three velocity components onto a denser unstructured mesh using a Gaussian–Kernel approach before computing spatial derivatives. This procedure regularizes velocity field (reducing spurious velocity divergence) and improves the numerical stability of gradient-based estimates, consistent with established preprocessing strategies implemented for deriving wall shear stress and vorticity from 4D flow MRI data [28, 82, 83]. Third, the cohort sizes were unequal (*n* = 41 for TAV and *n* = 19 for BAV), which may affect statistical power. Fourth, the limited number of BAV individuals did not permit stratification by fusion subtype, despite its known influence on flow eccentricity and AAo helical flow [36, 45, 76]. Future studies with larger and better-stratified cohorts are needed to validate our findings and to specifically assess the influence of different BAV fusion types on AAo helicity. Moreover, as this investigation was cross-sectional, longitudinal studies will be essential to definitively clarify the role of helicity in the onset and progression of BAV-related aortopathy. Finally, this study was limited to large-scale AAo hemodynamics, as the 4D flow MRI sequences required for turbulence-based quantities were not available. Expanding future in vivo investigations to include small-scale hemodynamics may provide further insights into the role of helical blood flow in delaying transition to turbulence in aorta [20].

## Conclusion

This study shows that aortic valve phenotype critically shapes helical flow in the ascending aorta through phenotype-specific anatomical and hemodynamic determinants. In TAV subjects, coherent helical fluid structures are primarily driven by pulsatile flow and vessel anatomy. In contrast, BAV individuals exhibit eccentric jets that disrupt helicity intensity, even though they promote organization in helical fluid structures. This reduction in intensity may compromise the protective role of helical flow against adverse biological events, potentially contributing to BAV-related aortopathy. Notably, helical flow topology (quantified in terms of ${\langle |LNH|\rangle }_{T{V}_{{AAo}}}$) emerged as a robust hallmark of BAV phenotype when integrated with anatomical or conventional hemodynamic parameters. Overall, these findings deepen our mechanistic understanding of BAV-associated flow abnormalities and highlight helicity-based metrics as promising biomarkers for risk stratification and clinical management.

## Supplementary Information

Below is the link to the electronic supplementary material.

Supplementary file1 (DOCX 403 KB)

## Data Availability

The data that support the findings of this study are not publicly available due to privacy and ethical concerns. They are available upon reasonable request from the corresponding author.
