# Supplementary material for: Aortic Valve Phenotype Shapes Helical Flow in the Ascending Aorta: Insights from 4D Flow MRI
Source: Ann Biomed Eng. 2026 Apr 7;54(11):3726–42. doi: 10.1007/s10439-026-04088-8 (PMC13694507; doi:10.1007/s10439-026-04088-8)
Supplement: Supplementary file 1 [file 10439_2026_4088_MOESM1_ESM.docx]

- **Supplementary Data** -

***Aortic Valve Phenotype Shapes Helical Flow in the Ascending Aorta:***

***Insights from 4D Flow MRI***

Karol Calò^1^, Andrea Guala^2,3^, Valentina Mazzi^1^, Lydia Dux-Santoy^2^, José F. Rodriguéz Palomares^2,3,4^, Stefania Scarsoglio^1^, Luca Ridolfi^5^, David A. Steinman^6^, Diego Gallo^1^, Umberto Morbiducci*^1^

*^1^ Polito^BIO^Med Lab, Department of Mechanical and Aerospace Engineering, Politecnico di Torino, Turin, Italy*

*^2^ Vall d’Hebron Institut de Recerca, Barcelona, Spain*

*^3^ Biomedical Research Networking Center on Cardiovascular Diseases, Instituto de Salud Carlos III, Madrid, Spain*

*^4^ Department of Cardiology, Hospital Universitari Vall d’Hebron, Barcelona, Spain*

*^5^ Polito^BIO^Med Lab, Department of Environment, Land and Infrastructure Engineering, Politecnico di Torino, Turin, Italy*

*^6^ Department of Mechanical and Industrial Engineering, University of Toronto, Toronto, Canada*

***Address for correspondence:**

Umberto Morbiducci, Full Professor

Department of Mechanical and Aerospace Engineering, Politecnico di Torino

Corso Duca degli Abruzzi, 24

10129 Turin, Italy

Tel.: +39 011 0906882

Fax: +39 011 5646999

E-mail: [umberto.morbiducci@polito.it](mailto:umberto.morbiducci@polito.it)

**Supplementary Methods and Results**

We conducted a preliminary sensitivity analysis to quantify the effect of velocity field interpolation settings on helicity-based quantities and to guide the selection of Gaussian kernel size and unstructured mesh resolution. Using a representative TAV subject, we assessed the dependence of the quantities in **Table 1** (calculated in the AAo) on (i) the Gaussian kernel radius *r* and (ii) the average element size *l* of the interpolation mesh. Results are summarized in **Tables S1** and **S2**.

Overall, helicity-based quantities showed limited sensitivity to both parameters. Variations were larger for changes in mesh resolution (up to ~29% relative to the finest mesh; **Table S2**) than for changes in kernel radius (up to ~6% relative to the smallest kernel; **Table S1**). These findings indicate that the estimation of the investigated helicity-based quantities is generally robust. In particular, $\left\langle|LNH| \right\rangle_{TV_{AAo}}$ was essentially insensitive to reasonable variations in interpolation and discretization settings.

Based on these results, we selected *r* = 3 mm (yielding ≤ ~3% variation compared with *r* = 2.5 mm) and *l* = 1.6 mm (yielding ≤ ~11% variation compared with *l* =0.8 mm). This choice provides a suitable trade-off between numerical stability of helicity-based quantities estimation and spatial resolution.

**Table S1 Effect of Gaussian kernel size *r* on helicity-based quantities estimation**

|  | *r* = 2.5 mm | *r* = 3 mm  (%diff with r = 2.5 mm) | *r* = 3.5 mm  (%diff with r = 2.5 mm) |
| --- | --- | --- | --- |
| ***h_1_* [m/s^2^]** | -0.518 | -0.515 (-0.56%) | -0.504 (-2.89%) |
| ***h_2_* [m/s^2^]** | 4.208 | 4.080 (-3.01%) | 3.940 (-6.34%) |
| ***h_3_*** | -0.123 | -0.126 (2.44%) | -0.128 (4.06%) |
| ***h_4_*** | 0.123 | 0.126 (2.44%) | 0.128 (4.06%) |
| $\left\langle\boldsymbol{\vert LNH\vert} \right\rangle_{\boldsymbol{T}\boldsymbol{V}_{\boldsymbol{AAo}}}$ | 0.458 | 0.457 (-0.22%) | 0.457 (-0.22%) |

**Table S2 Effect of interpolation mesh size *l* on helicity-based quantities estimation**

|  | *l* = 0.8 mm | *l* = 1.6 mm  (%diff with *l* = 0.8 mm) | *l* = 2.4 mm  (%diff with *l* = 0.8 mm) |
| --- | --- | --- | --- |
| ***h_1_* [m/s^2^]** | -0.580 | -0.515 (-11.22%) | -0.407 (-29.75%) |
| ***h_2_* [m/s^2^]** | 4.291 | 4.080 (-4.91%) | 3.906 (-8.98%) |
| ***h_3_*** | -0.135 | -0.126 (-6.67%) | -0.104 (-22.96%) |
| ***h_4_*** | 0.135 | 0.126 (-6.67%) | 0.104 (-22.96%) |
| $\left\langle\boldsymbol{\vert LNH\vert} \right\rangle_{\boldsymbol{T}\boldsymbol{V}_{\boldsymbol{AAo}}}$ | 0.457 | 0.457 (0.00%) | 0.456 (-0.22%) |

**Supplementary figure**

**Fig. S1.** Boxplots comparing helicity-based quantities in the TAV and BAV groups, obtained by analyzing systole and diastole as separate observation windows. In the plots, the median is indicated by the black line, and the box indicates the interquartile range. Differences between the TAV and BAV groups of subjects in systole and diastole were assessed using Mann-Whitney U tests. SYS: systole; DIAS: diastole
